# Effectiveness of evidence-based fall prevention programs to reduce loneliness in the United States

**DOI:** 10.3389/fpubh.2024.1459225

**Published:** 2024-09-06

**Authors:** Matthew Lee Smith, Gang Han

**Affiliations:** ^1^Department of Health Behavior, School of Public Health, Texas A&M University, College Station, TX, United States; ^2^Center for Community Health and Aging, Texas A&M University, College Station, TX, United States; ^3^Department of Epidemiology and Biostatistics, School of Public Health, Texas A&M University, College Station, TX, United States

**Keywords:** fall prevention, loneliness, evidence-based program, older adult, evaluation

## Abstract

**Introduction:**

Falls are associated with activity limitations and injuries among older adults. An estimated 25% of older adults fall each year, and over 40% of older adults report they are lonely. Small group, evidence-based fall prevention programs are widely available in the United States and may be a strategy to improve social connectedness within our aging population. The purpose of this study was to identify the effectiveness of evidence-based fall prevention programs to reduce loneliness among older adults. Administration for Community Living (ACL) grantee data were collected in a national repository.

**Methods:**

Data were analyzed from 12,944 participants across 12 fall prevention programs (e.g., A Matter of Balance, Stepping On, Tai Ji Quan, Otago Exercise Program, Bingocize) between January 2021 and July 2023. To assess loneliness, participants were asked, “how often do you feel lonely or isolated?” The response choices for this single 5-point item ranged from “never” to “always.” A linear mixed-effects multivariable regression, with program type included as a random effect, was fitted to assess changes in loneliness before and after fall prevention workshops. The model controlled for program type and delivery site type as well as participants’ age, sex, ethnicity, race, education, living alone, number of chronic conditions, number of falls in the three months preceding baseline, and workshop delivery site type and attendance.

**Results:**

Significant reductions in loneliness scores were observed from baseline to post-workshop (*p* < 0.001), which were more pronounced among participants with more frequent baseline loneliness (*p* < 0.001). Participants who attended more workshop sessions reported reduced loneliness at post-workshop (*p* = 0.028). From baseline to post-workshop, loneliness increased among participants who lived alone (*p* < 0.001) and reported two or more falls in the three months preceding baseline (*p* =0.002). From baseline to post-workshop, compared to White participants, increased loneliness was observed among Black (*p* = 0.040), and Asian (*p* < 0.001) participants. Participants with more chronic conditions reported more loneliness from baseline to post-workshop (*p* = 0.004). Relative to participants who attended workshops at senior centers, increased loneliness was observed among participants who attended workshops at residential facilities (*p* = 0.034) and educational institutions (*p* = 0.035).

**Discussion:**

Findings expand our understanding about the benefits of small-group fall prevention workshops to reduce loneliness among older participants. Results suggest that disease profiles, living alone, fall history, and workshop location (and attendee dynamic) may impede social connection among some participants. Beyond small group activities, purposive strategies should be embedded within fall prevention programs to foster meaningful interactions and a sense of belonging between participants. Other social connection programs, services, and resources may complement fall prevention programming to reduce loneliness.

## 1 Introduction

With about one-in-four older adults ages 65 years and older falling each year (1), falls remain a sizable public health issue in the United States. A fall is defined as “an event which results in a person coming to rest inadvertently on the ground or floor or other lower level” (2). Falls are the leading cause of unintentional injuries and deaths among older adults (3), and older adults who fall have an increased risk of negative health consequences including recurrent falls, diminished mobility, and loss of independence (1, 4). To prevent falls and their costly ramifications, over a dozen evidence-based fall prevention programs (EBFPP) are delivered nationwide through the aging services network.

A robust and expansive delivery infrastructure has been created in the United States to implement EBFPP through the aging services network, which is supported by the Administration for Community Living, Older Americans Act (Title III-D), the Centers for Disease Control and Prevention, and other local, state, and private funding sources (5–8). Each EBFPP differs in its content, format, intensity, and intended audience (9), yet each was purposively developed to directly benefit older adults by addressing one or more fall-related risk factors (e.g., fear of falling, efficacy to prevent falls, lower limb strength, and balance). The effectiveness of EBFPP was demonstrated in experimental trials prior to being translated for grand-scale dissemination in community settings (10). Examples of EBFPP include programs such as A Matter of Balance (11), Bingocize (12), Stepping On (13), and Tai Ji Quan (14). Most EBFPP are delivered in small-group, in-person workshops in diverse community settings including senior centers, healthcare organizations, residential facilities, and faith-based organizations (7, 8). While these programs have directly benefited hundreds of thousands of older adults to address fall-related risk, the process-driven nature of these programs may provide other indirect benefits to older adult participants. For example, the small-group, in-person nature of EBFPP workshops may promote social connection because they facilitate meaningful interactions and bonding among older adults by gathering participants for common purposes, facilitating interactive exchanges, and creating opportunities for frequent engagement with peers and trained lay leaders for multiple consecutive weeks.

In 2023, the United States Surgeon General declared loneliness as an epidemic facing all Americans, including older adults (15). Loneliness can be defined as a subjective measure of feeling isolated or the distress of having inadequate meaningful connections with others (15–18). It is estimated that between 20 and 40% of older adults have moderate to severe loneliness (19–21), which can impact multiple aspects of their health and wellbeing. Older adults who are lonely have an increased risk of physical and mental health issues, suicidal ideation, and premature mortality (22–26). Therefore, amidst emerging evidence of interventions to address loneliness among older adults (27, 28), efforts are needed to engage older adults in more programs and services that can expand and strengthen relationships, promote meaningful interactions, and facilitate bonding and social support.

Loneliness may be bidirectionally associated with falling (29). Older adults who are lonely may restrict their social activities and receive limited functional support from others, which may increase their risk for falling. Conversely, if an older adult has an injurious fall, their loneliness may be exacerbated because of physical isolation due to hospitalization or changes in mobility (e.g., difficulty walking, inability to drive). Given the widespread recognition of the deleterious effects of loneliness on older adult health (15, 16), recent efforts have attempted to identify the indirect benefits of existing evidence-based programs (EBP) originally developed for other purposes (30–32). These investigations have revealed the effectiveness of EBP to reduce loneliness, increase social support, and promote social connection. Therefore, it is important to understand the potential indirect benefits of EBFPP because of their grand-scale availability and accessibility through the aging services network in the United States. In this context, the purposes of this study were to: (1) identify the frequency of feeling lonely among participants enrolled in EBFPP; and (2) assess the effectiveness of EBFPP to reduce loneliness among older adults.

## 2 Methods

### 2.1 Participants and procedures

Data for this study were obtained from the Healthy Aging Program Integrated Database (HAPID®), a national repository funded by the National Falls Prevention and Chronic Disease Self-Management Program Resource Centers to support efforts by chronic disease self-management education (CDSME) and EBFPP grantees across the United States (33, 34). As part of their funding agreements, grantees funded by the Administration for Community Living (ACL) through the Prevention and Public Health Fund (PPHF) were required to use uniform data collection forms and enter data into HAPID®. The data contained within the repository includes information about workshops, participants (at baseline and post-workshop), attendance records, and organizations serving as host and delivery sites (7, 8, 35). Data used for this study included efforts from 41 grantees spanning 30 states from January 2021 and July 2023. Data were collected locally by workshop leaders and organizations hosting programs, which was then entered in a centralized or de-centralized manner by each grantee. It is important to note that the delivery of EBFPP through the network of aging and healthcare organizations across the United States is not limited to these ACL grantees; rather, funding for EBFPP may be from the Older Americans Act Title III-D, the Centers for Disease Control and Prevention Arthritis Program, or other local, state, and private funding sources. Neither these data nor efforts. Institutional Review Board approval was granted by Texas A&M University (#2020-1244) for this secondary, de-identified data analysis from this national repository.

### 2.2 Measures

#### 2.2.1 Dependent variable

Loneliness was assessed at baseline and post-workshop using an identical single five-point Likert-type item. Participants were asked “How often do you feel lonely or isolated?” Response choices were “never” (scored 1), “rarely” (scored 2), “sometimes” (scored 3), “often” (scored 4), and “always” (scored 5).

#### 2.2.2 Health indicators

Participants were asked to report if a healthcare professional ever told them that they had a chronic health condition from a list of 20 disease types (e.g., arthritis, breathing/lung disease, cancer, chronic pain, depression, diabetes, heart disease, kidney disease, and Parkinson’s disease). The number of chronic conditions endorsed by each participant were summed to create a count variable, which was used continuously in analyses. At baseline, participants were asked to report the number of falls they experienced in the 3 months preceding the EBFPP. Participants’ responses were collapsed to create a three-category variable with options of “no falls,” “one fall,” and “two or more falls.”

#### 2.2.3 Program information

Administrative records were used to identify characteristics of the workshops in which participants enrolled. The program type was documented, which included 12 EBFPP [i.e., A Matter of Balance, Bingocize, Stay Active & Independent for Life (SAIL), Tai Chi for Arthritis, Stepping On, Tai Ji Quan, Healthy Steps for Older Adults, CAPABLE, Fit & Strong!, Tai Chi Prime, YMCA Moving for Better Balance, and Otago Exercise Program]. The delivery site type where workshops were hosted was included (i.e., senior center, community center, workplace, residential facility, healthcare organization, faith-based organization, educational institution, and government organization). Participants reported whether they were referred to attend the EBFPP by a healthcare provider (i.e., “no” or “yes”). Participants’ attendance in the EBFPP workshop were recorded and reported. Because EBFPP typically include a different number of workshop sessions, a ratio was calculated to uniformly identify the proportion of workshop sessions participants attended (i.e., ranging from 0 to 100% of workshop sessions). Successful completion of EBFPP workshops (i.e., attending the recommended number of workshop sessions to suggest the proper intervention dose was received), as defined by each program developer, respectively, was calculated. However, successful workshop completion was only reported descriptively in the current study.

#### 2.2.4 Sociodemographics

Participant characteristics included age, sex (i.e., “female,” “male,” or “prefer not to report”), Hispanic ethnicity (i.e., “no” or “yes”), race (i.e., “White,” “Black or African American,” “Asian or Pacific Islander,” “other or multiple races,” or “unknown”), education level (i.e., “high school education or less,” “some college or 2-year degree,” “college graduate or more,” or “unknown”), and living alone (i.e., “no” or “yes”).

### 2.3 Statistical analyses

All analyses were performed using SAS 9.4. Data were initially analyzed from 20,539 older adults who enrolled in an EBFPP between May 2021 and June 2023. Based on study purposes, participants with matched baseline and post-workshop loneliness data were prioritized for repeated measures analyses. Sample characteristics (i.e., participant and program characteristics) were compared using chi-square tests and two-tailed *t*-tests to identify differences between participants with (*n* = 12,944) and without (*n* = 7,595) post-workshop data (tables not reported). Then, only using data from the 12,944 participants with matched baseline and post-workshop loneliness data, chi-square tests and two-tailed *t*-tests were used to identify differences between participants’ loneliness levels at baseline. Medians and interquartile rankings (IQR) are reported for continuous variables. A linear mixed-effects multivariable regression was fitted to assess changes in loneliness before and after fall prevention workshops. Program type was included in the model as a random effect. The model also controlled for participants’ age, sex, ethnicity, race, education, living alone, number of chronic conditions, number of falls in the 3 months preceding baseline, workshop delivery site type, and workshop attendance. For all analyses, *p* values <0.05 were used to identify statistical significance.

## 3 Results

When examining all available baseline data (*n* = 20,539), most participants reported “never” (32%), “rarely” (36%), and “sometimes” (27%) feeling lonely or isolated, compared to smaller proportions who reported “often” (4%) and “always” (1%) feeling lonely or isolated. When comparing participants with and without matched baseline and post-workshop loneliness data, those with matched data reported more frequent loneliness at baseline. Compared to those with only baseline loneliness data, on average, participants with matched loneliness data were older and had fewer chronic conditions. A significantly larger proportion of participants with matched loneliness data were non-Hispanic, non-White, and less educated. Compared to those with only baseline data, a smaller proportion of participants with matched baseline and post-workshop data reported one or more falls in the 3 months prior to enrolling in the EBFPP. Relative to those without follow-up loneliness data, a significantly smaller proportion of participants with matched data were referred to the EBFPP by a healthcare professional. Larger proportions of participants with follow-up loneliness data attended A Matter of Balance, Bingocize, SAIL, and Stepping On, whereas smaller proportions of participants with follow-up data attended Tai Chi for Arthritis and Tai Ji Quan. Larger proportions of participants with follow-up loneliness data attended workshops at community centers and residential facilities, whereas smaller proportions of participants attended workshops at workplaces, healthcare organizations, and educational institutions. On average, participants with matched loneliness data attended larger percentages of workshop sessions, with significantly larger proportions successfully completing workshops.

Among those with matched baseline and post-workshop loneliness data (*n* = 12,944), Table 1 reports participant-related variables, which are compared by participants’ baseline loneliness levels. At baseline, most participants reported “never” (32%), “rarely” (36%), and “sometimes” (28%) feeling lonely or isolated, compared to smaller proportions who reported “often” (3%) and “always” (1%) reporting feeling lonely or isolated. The median age of participants at baseline was age 75 years (IQR: 70, 81). About 83% of participants were female, 94% were non-Hispanic, 77% were White, 10% were Black or African American, and 6% were Asian or Pacific Islander. Most participants reported having a college degree (41%) or attending some college or having a 2-year degree (28%). The median number of self-reported chronic conditions was 3 (IQR: 1, 4). About 46% of participants lived alone. In the 3 months prior to enrolling in the EBFPP, 78% of participants reported no falls, 14% reported one fall, and 8% reported two or more falls.

**Table 1:** Participant-related variables by baseline loneliness level.

	Total (*n* = 12,944)	Never (*n* = 4,124)	Rarely (*n* = 4,680)	Sometimes (*n* = 3,640)	Often (*n* = 423)	Always (*n* = 77)	*p*-value
Age	75 [70, 81]	75 [69,81]	75 [70,81]	75 [70,81]	74 [69,80]	72 [66,77]	<0.001
Sex							<0.001
Female	10,558 (83%)	3,192 (79%)	3,866 (84%)	3,085 (86%)	357 (86%)	58 (77%)	
Male	2,104 (17%)	831 (21%)	725 (16%)	476 (13%)	55 (13%)	17 (23%)	
Prefer not to report	33 (0%)	14 (0%)	4 (0%)	13 (0%)	2 (0%)		
Hispanic ethnicity							<0.001
No	11,335 (94%)	3,616 (95%)	4,170 (96%)	3,130 (92%)	355 (89%)	64 (93%)	
Yes	705 (6%)	197 (5%)	187 (4%)	271 (8%)	45 (11%)	5 (7%)	
Race							<0.001
White	9,962 (77%)	3,178 (77%)	3,803 (81%)	2,630 (72%)	305 (72%)	46 (60%)	
Black or African American	1,358 (10%)	536 (13%)	416 (9%)	370 (10%)	26 (6%)	10 (13%)	
Asian or Pacific Islander	761 (6%)	137 (3%)	200 (4%)	372 (10%)	38 (9%)	14 (18%)	
Other or Multiple races	863 (7%)	273 (7%)	261 (6%)	268 (7%)	54 (13%)	7 (9%)	
Education level							<0.001
High school education or less	3,470 (27%)	1,076 (26%)	995 (21%)	1,230 (34%)	136 (32%)	33 (43%)	
Some college or 2-year degree	3,587 (28%)	1,166 (28%)	1,382 (30%)	914 (25%)	106 (25%)	19 (25%)	
College graduate or more	5,303 (41%)	1,691 (41%)	2,121 (45%)	1,313 (36%)	164 (39%)	14 (18%)	
Unknown	584 (5%)	191 (5%)	182 (4%)	183 (5%)	17 (4%)	11 (14%)	
Number of chronic conditions	3 [1,4]	2 [1,4]	3 [1,4]	3 [2,5]	4 [2,6]	5 [2,7]	<0.001
Live alone							<0.001
No	6,878 (54%)	2,716 (67%)	2,532 (55%)	1,464 (41%)	144 (34%)	22 (29%)	
Yes	5,844 (46%)	1,334 (33%)	2058 (45%)	2,124 (59%)	274 (66%)	54 (71%)	
Fall history at baseline							<0.001
No falls	9,224 (78%)	3,067 (83%)	3,403 (80%)	2,466 (74%)	249 (65%)	39 (53%)	
One fall	1,600 (14%)	420 (11%)	580 (14%)	516 (15%)	71 (18%)	13 (18%)	
Two or more falls	962 (8%)	229 (6%)	279 (7%)	367 (11%)	66 (17%)	21 (29%)	

When comparing participant-related variables by baseline loneliness, significantly higher levels of loneliness were reported by younger participants (*p* < 0.001) and those with more chronic conditions (*p* < 0.001). Larger proportions of participants who were non-Hispanic (*p* < 0.001), non-White (*p* < 0.001), and those with lower education levels (*p* < 0.001) reported more frequent loneliness at baseline. A larger proportion of participants who lived alone (*p* < 0.001) and reported falling once more in the 3 months before enrolling in the EBFPP (*p* < 0.001) reported higher levels of loneliness. A significantly larger proportion of men reported either “never” or “always” feeling lonely at baseline (*p* < 0.001).

Table 2 reports program-related variables, which are compared by participants’ baseline loneliness levels. Thirteen percent of participants were referred to EBFPP by a healthcare professional. The most attended programs were A Matter of Balance (32%), Bingocize (17%), SAIL (17%), Tai Chi for Arthritis (16%), Stepping On (8%), and Tai Ji Quan (8%). EBFPP workshops were most attended at senior centers (28%), community centers (18%), workplaces (16%), and residential facilities (14%). About 71% of participants successfully completed EBFPP workshops in which they were enrolled, with an average attendance of 79% of offered workshops.

**Table 2:** Program-related variables by baseline loneliness level.

	Total (*n* = 12,944)	Never (*n* = 4,124)	Rarely (*n* = 4,680)	Sometimes (*n* = 3,640)	Often (*n* = 423)	Always (*n* = 77)	*p*-value
Referred by healthcare professional							<0.001
No	10,566 (87%)	3,417 (88%)	3,910 (89%)	2,878 (84%)	309 (77%)	52 (70%)	
Yes	1,592 (13%)	445 (12%)	499 (11%)	533 (16%)	93 (23%)	22 (30%)	
Program name							<0.001
A matter of balance	4,169 (32%)	1,206 (29%)	1,497 (32%)	1,264 (35%)	165 (39%)	37 (48%)	
Bingocize	2,162 (17%)	695 (17%)	588 (13%)	761 (21%)	98 (23%)	20 (26%)	
SAIL	2,229 (17%)	818 (20%)	909 (19%)	459 (13%)	41 (10%)	2 (3%)	
Tai Chi for Arthritis	2072 (16%)	715 (17%)	846 (18%)	469 (13%)	37 (9%)	5 (6%)	
Stepping On	1,013 (8%)	305 (7%)	380 (8%)	296 (8%)	30 (7%)	2 (3%)	
Tai Ji Quan	1,005 (8%)	278 (7%)	355 (8%)	319 (9%)	44 (10%)	9 (12%)	
Healthy Steps for Older Adults	161 (1%)	62 (2%)	57 (1%)	40 (1%)	2 (0%)	—	
CAPABLE	29 (0%)	6 (0%)	7 (0%)	9 (0%)	5 (1%)	2 (3%)	
Fit & Strong!	62 (0%)	23 (1%)	26 (1%)	12 (0%)	1 (0%)	—	
Tai Chi Prime	17 (0%)	4 (0%)	6 (0%)	7 (0%)	—	—	
YMCA Moving for Better Balance	17 (0%)	9 (0%)	5 (0%)	3 (0%)	—	—	
Otago Exercise Program	8 (0%)	3 (0%)	4 (0%)	1 (0%)	—	—	
Delivery site type							<0.001
Senior Center	3,598 (28%)	1,190 (29%)	1,235 (26%)	1,005 (28%)	147 (35%)	21 (27%)	
Community Center	2,329 (18%)	754 (18%)	805 (17%)	684 (19%)	69 (16%)	17 (22%)	
Workplace	2024 (16%)	715 (17%)	780 (17%)	472 (13%)	49 (12%)	8 (10%)	
Residential Facility	1833 (14%)	529 (13%)	567 (12%)	653 (18%)	65 (15%)	19 (25%)	
Healthcare Organization	1,100 (9%)	331 (8%)	447 (10%)	271 (7%)	45 (11%)	6 (8%)	
Faith-Based Organization	1,051 (8%)	320 (8%)	455 (10%)	251 (7%)	22 (5%)	3 (4%)	
Educational Institution	635 (5%)	174 (4%)	258 (6%)	186 (5%)	16 (4%)	1 (1%)	
Government Organization	366 (3%)	111 (3%)	132 (3%)	112 (3%)	9 (2%)	2 (3%)	
Proportion of workshop sessions attended	85.7 [70.8,100]	85 [68.8, 95.8]	85.7 [70.7,100]	87.5 [71.4,100]	87.5 [75,100]	87.5 [79.2,100]	<0.001

When comparing program-related variables by baseline loneliness, a significantly larger proportion of participants referred to attend an EBFPP by a healthcare professional reported more frequent loneliness (*p* < 0.001). Larger proportions of participants who attended A Matter of Balance, Bingocize and Tai Ji Quan, reported more frequent loneliness at baseline, whereas smaller proportions of participants who attended SAIL, Stepping On, and Tai Chi for Arthritis reported less frequent loneliness at baseline (*p* < 0.001). Larger proportions of participants who attended EBFPP at residential facilities reported more frequent loneliness at baseline, whereas smaller proportions of participants who attended EBFPP at workplaces and faith-based organizations reported less frequent loneliness at baseline (*p* < 0.001). On average, participants who attended larger percentages of workshop sessions reported more frequent loneliness at baseline (*p* < 0.001).

On average, from baseline to post-workshop, participants reported a significant reduction in loneliness (*t* = −4.5, *p* < 0.001); 19.3% of participants reported less frequent loneliness, 63.1% stayed the same, and 17.6% reported more frequent loneliness. Table 3 reports the linear mixed-effects multivariable regression adjusting for participant- and program-related variables. Relative to participants who reported “never” experiencing loneliness at baseline, participants who reported experiencing loneliness “rarely” (Estimate = −0.351, *p* < 0.001), “sometimes” (Estimate = −1.341, *p* < 0.001), and “often” (Estimate = −2.272, *p* < 0.001) reported significantly less loneliness at post-workshop, respectively. Participants of older ages (Estimate = −0.003, *p* = 0.006) and who attended larger proportions of EBFPP workshops sessions (Estimate = −0.001, *p* = 0.028) reported significantly less loneliness at post-workshop, respectively. Significant reductions in loneliness were observed among Hispanic participants, compared to their non-Hispanic counterparts (Estimate = −0.247, *p* < 0.001). Relative to White participants, significant increases in loneliness were observed among participants who were Black or African American (Estimate = 0.068, *p* = 0.040), Asian or Pacific Islander (Estimate = 0.212, *p* < 0.001), and other or multiple races (Estimate = 0.101, *p* = 0.018), respectively. Having more chronic conditions was associated with significant increases in loneliness from baseline to post-workshop (Estimate = 0.012, *p* = 0.004). Significant increases in loneliness were reported among participants who lived alone compared to those who lived with others (Estimate = 0.097, *p* < 0.001). Compared to participants reporting no falls 3 months prior to enrolling in EBFPP, those who reported two or more falls reported significant increases in loneliness (Estimate = 0.110, *p* = 0.002). Relative to participants who attended EBFPP workshops at senior centers, significant increases in loneliness were observed among those who attended workshops at residential facilities (Estimate = 0.067, *p* = 0.034) and educational institutions (Estimate = 0.094, *p* = 0.035), respectively.

**Table 3:** Factors associated with changes in loneliness over time.

	Estimate	S.E.	*p*-value
Baseline loneliness: Never	—	—	—
Baseline loneliness: Rarely	−0.351	0.023	<0.001
Baseline loneliness: Sometimes	−1.341	0.025	<0.001
Baseline loneliness: Often	−0.081	0.054	0.133
Baseline loneliness: Always	−2.272	0.122	<0.001
Age	−0.003	0.001	0.006
Sex: Female	—	—	—
Sex: Male	−0.022	0.025	0.383
Sex: Prefer not to reply	−0.190	0.206	0.357
Hispanic: No	—	—	—
Hispanic: Yes	−0.247	0.042	<0.001
Race: White	—	—	—
Race: Black	0.068	0.033	0.040
Race: Asian	0.212	0.042	<0.001
Race: Other or multiple races	0.101	0.042	0.018
Education: High school or less	—	—	—
Education: Some college or 2-year degree	−0.021	0.026	0.426
Education: College graduate or more	−0.024	0.025	0.332
Education: Unknown	−0.109	0.055	0.047
Number of chronic conditions	0.012	0.004	0.004
Live alone: No	—	—	—
Live alone: Yes	0.097	0.020	<0.001
Baseline falls: No falls	—	—	—
Baseline falls: One fall	0.023	0.028	0.404
Baseline falls: Two or more falls	0.110	0.035	0.002
Healthcare referral: No	—	—	—
Healthcare referral: Yes	0.036	0.028	0.198
Delivery site: Senior Center	—	—	—
Delivery site: Community Center	−0.026	0.029	0.382
Delivery site: Workplace	0.001	0.031	0.985
Delivery site: Residential Facility	0.067	0.032	0.034
Delivery site: Healthcare Organization	0.066	0.038	0.078
Delivery site: Faith-Based Organization	−0.016	0.037	0.676
Delivery site: Educational Institution	0.094	0.044	0.035
Delivery site: Government Organization	0.007	0.061	0.914
Proportion of workshop sessions attended	−0.001	0.000	0.028

## 4 Discussion

This study aimed to identify the indirect benefits of small-group EBFPP to reduce feelings of loneliness among older adult participants. At baseline, large proportions of participants reported lower levels of feeling lonely or isolated (i.e., 32% reporting “never” and 36% reporting “rarely”), which is lower than the reported prevalence among older adults nationwide (19–21). Regardless, analyses showed a modest yet significant reduction in loneliness across participants from baseline to post-workshop, which adds to the current literature regarding the indirect benefits of interventions to address aspects of social disconnectedness despite being developed for other purposes (30–32). The interactive, in-person EBFPP sessions held over a series of consecutive weeks gives participants opportunities to engage with one another, and trained lay leaders, to brainstorm and problem-solve for a common purpose of preventing falls. Gathering groups of older adults for programming may expand social networks, and the group dynamic developed over time may facilitate social bonding and social support, which addresses structural and functional elements of social connectedness (15, 30). The current study also showed a dose–response in that participants with higher EBFPP attendance exhibited greater reductions in loneliness, further supporting the indirect benefits of small-group cohesion for those who engage more with the intervention.

In the current study, loneliness-related benefits differed by participant and program characteristics. Intuitively, participants who reported higher levels of loneliness at baseline were more likely to report reductions in loneliness post-workshop. Participants of younger ages were more likely to report reductions in loneliness relative, which may be associated with these participants entering EBFPP with higher levels of loneliness, attending more workshop sessions, and/or attending workshops in certain settings. For example, in the current study, participants who attended workshops in residential facilities were less likely to report reductions in loneliness post-workshop, and participants residing in these settings tend to be older and have more co-morbidities and complex health conditions (e.g., more falls preceding the workshop) that may hinder workshop attendance (36). Additionally, participants who attended more workshop sessions reported lower loneliness levels post-workshop, which aligns with previous findings from Chronic Disease Self-Management Education (CDSME) programs (30) and highlights the need for program implementers and community sites to focus on participant retention to ensure adequate intervention dose.

A recent systematic review identified no significant differences in loneliness prevalence or severity across ethnic groups in the United States (37). Yet, in the current study, Hispanic participants reported greater lower loneliness levels post-workshop relative to their non-Hispanic counterparts. Conversely, compared to White participants, analyses revealed that participants who identified as Black, Asian, or another race reported higher loneliness levels post-workshop, respectively. These findings align with previous studies that identified greater prevalence rates of loneliness among underserved and minoritized groups (38–40). Changes in loneliness among these participant subgroups, for better or worse, may be attributed to an interplay of factors including the dynamics resulting from the composition of small-group workshop attendees and the communities and settings in which EBFPP were hosted. For example, many evidence-based programs for older adults, and EBFPP specifically, have been culturally tailored for Hispanic communities and are offered in Spanish (9, 41). Culturally-tailored workshops may foster stronger group cohesion in that participants are more likely to share community and cultural commonalities (42–44). Therefore, to complement efforts examining racial/ethnic diversity in EBFPP (45) and enhance intervention engagement and group cohesion among populations traditionally underserved by EBFPP, efforts are needed to purposively adapt EBFPP and other evidence-based programs for culturally-and linguistically-diverse subgroups.

Living alone has been identified as a risk factor for social isolation and loneliness because it may be indicative of a limited social infrastructure and/or infrequent interactions with others (46, 47). In the current study, participants who lived alone reported higher levels of loneliness at baseline and post-intervention. These findings may reflect known risk factors for older adults who live alone, suggesting these individuals may lack desired levels of in-home social interactions or the social support needed to attend EBFPP workshop sessions (e.g., motivational encouragement, transportation). Because living alone is not necessarily indicative of loneliness (48), additional efforts should examine the personal and workshop characteristics associated with EBFPP participation and successful completion among participants who live alone.

This study had limitations, which warrant acknowledgement. First, the analytic sample was relatively homogenous, with the majority representing non-Hispanic, White females. Although the sociodemographics of this sample mirror those from previous grand-scale studies of grant-funded EBFPP (7, 8, 45), the ability for communities to offer EBFPP using Title III-D funding suggests this sample may not be representative of all EBFPP program participants (i.e., from grant-and non-grant-funded community initiatives). Additional efforts are needed to assess loneliness among a more diverse array of EBFPP participants. Second, participation in EBFPP is voluntary, thus there was no comparison group and older adults who elected to enroll in such programming may have differed from those who did not. This self-selection bias may limit the generalizability of study findings to the greater older adult population. Third, loneliness data were self-reported using a single item. Self-reported loneliness may be subject to social desirability bias and underreporting, especially considering the stigma surrounding loneliness in the United States (49, 50). The use of a single item to measure loneliness limited the robustness of understanding participants’ loneliness and detecting its change over time. More specifically, in the current study, a small proportion of participants reported high levels of loneliness at baseline (i.e., 3% reporting “often” and 1% reporting “always”), which may have introduced a “floor effect” for the intervention where participants were unable to show improvement from baseline to post-workshop. It is recommended that future studies use other validated scales to better understand the indirect benefits of EBFPP on loneliness. Suggested scales include the Revised UCLA Loneliness Scale (51, 52), de Jong Gierveld Loneliness Scale (53, 54), Campaign to End Loneliness Measurement Tool (55), the Upstream Social Interaction Risk Scale (56, 57), or others (58). Fourth, there was substantial missing post-workshop data, which reduced the analytic sample size by ~37% relative to available baseline data. While missing data is common in grand-scale, community-based implementations of EBFPP (59), systematic deficiencies in data collection and reporting may result in underrepresenting participants with certain characteristics (e.g., sex, race, low income) or from certain settings (e.g., rural). Because participants were not required to complete forms to attend workshops, efforts are needed to improve data collection among community-based organizations through technical assistance, training, and incentives for data collection fidelity. Fifth, data were analyzed from baseline to post-workshop across 12 EBFPP with varying workshop durations (i.e., number of weeks, time per sessions), formats (e.g., group size, lay leader role), and activities (e.g., education-based, physical activity-based). This may have impacted our ability to identify the nuances of program-specific effectiveness on loneliness (e.g., changes in loneliness may not be observed within workshops with shorter durations or limited peer interaction). Additional program-specific evaluations are needed to assess their indirect benefits on loneliness.

Despite these possible shortcomings, findings from this nationwide evaluation of EBFPP highlight their potential to reduce loneliness among older adults. This study builds upon the evidence related to the indirect benefits of EBP to address issues of social connectedness among older adults (30–32). The benefits of EBP generally, and EFFPP specifically, to address loneliness may be more process-drive than content-driven because small-group, in-person workshops gather older adults for common purposes, facilitate solution-oriented interactions and activities, and enable frequent engagement with peers and trained lay leaders for multiple consecutive weeks. As such, efforts are needed to diversify the recruitment of participants and workshop delivery locations to ensure representation from traditionally underserved population sub-groups (60) who may benefit from the direct and indirect benefits of the intervention. Ongoing efforts are needed to support the aging services network to grow and sustain the infrastructure necessary to offer EBFPP nationwide. Beyond the direct indirect benefits of EBFPP for participants, the cross-sectoral collaboration and coordination necessary across the aging services network to deliver EBFPP in a given community shows the promise of these initiatives as societal strategies to reduce silos and promote social connection among older adults (61).

Opportunities are available to complement existing EBFPP with additional elements to bolster their impact on loneliness and social disconnectedness by fostering meaningful interactions and a sense of belonging between participants. For example, program activities may be altered to incorporate more interactive peer-to-peer activities during workshop sessions or in addition to workshop sessions [e.g., session zeros (62), gatherings outside of session times during the workshop, gatherings transcending the official end of workshops]. Although additional activities can be added to EBFPP curricula, such modifications would need to carefully consider the additional costs and administrative burdens, which may not be reimbursable through the existing delivery infrastructure in the United States. EBFPP can also be accompanied by other social connection programming such as friendly calling, friendly visiting, or intergenerational interventions (27, 28). Beyond the small-group, in-person workshops, additional research is needed to examine the effectiveness of virtually-delivered EBFPP to address loneliness and social disconnectedness.

## 5 Conclusion

The purpose of this study was to identify the potential indirect benefits of EBFPP to reduce loneliness among older adults. Findings showed that baseline loneliness levels were low, yet a statistically significant reduction in loneliness were identified from baseline to post-workshop, on average. Participants who started workshops with higher loneliness levels and those who attended more workshop sessions reported lower loneliness at post-workshop. However, reductions in loneliness were not universal across all participant types, with some participants reporting higher loneliness levels at post-workshop (e.g., non-White, living alone, with a history of recurrent falling). Additional research is needed to examine the effectiveness of EBFPP separately to identify if they attract lonelier participants at baseline or have more pronounced impacts on loneliness over time (e.g., based on structure, activities, intensity, and duration). Taken together, findings suggest that EBFPP can reduce loneliness among older adult participants, which adds to the growing body of literature about the indirect benefits of evidence-based programs for older adults, which were developed for purposes other than social connection.

## Data Availability

The data analyzed in this study is subject to the following licenses/restrictions: data are available with requests to the National Council on Aging, with accompanying IRB approval and at data use agreement. Requests to access these datasets should be directed to https://www.ncoa.org/page/contact-us.
